# Modelling orexinergic system in ageing in the African turquoise killifish

**DOI:** 10.1007/s10522-025-10214-1

**Published:** 2025-03-14

**Authors:** Maria Raggio, Ivan Conte, Paolo de Girolamo, Livia D’Angelo

**Affiliations:** 1 Department of Veterinary Medicine and Animal Production, University of Naples Federico II, Naples, Italy; 2 Department of Biology, University of Naples Federico II, Naples, Italy

**Keywords:** Brain, Orexin, Healthy ageing, Neurodegenerative process, Teleost

## Abstract

The orexinergic system is anatomically and functionally conserved in almost all vertebrates, and the role in healthy ageing and age-associated diseases has been studied in mammals. Here, we review the main findings on the age-related regulation of orexinergic system in mammals, including human patients and highlights how the fish *Nothobranchius furzeri* serves as an exceptional model to spearhead research and unravel the intricate mechanisms underlying orexinergic regulation during ageing. The ageing brain of this teleost is characterized by the presence of neurodegenerative processes similar to those associated with human pathologies rather than those of healthy ageing. We present an in-depth summary and discussion on the groundbreaking advances in understanding the neuroanatomical organization of the orexinergic system, its pivotal role in mammalian and fish models, and its profound involvement in healthy ageing and age-associated diseases.

## Introduction

The orexinergic system comprises two neuropeptides, orexin A and B (OXA and OXB), also named hypocretin 1 and 2 (HCRT), which are generated by the cleavage of the precursor protein, prepro-orexin (PPO) (Sakurai et al. 1998; de Lecea et al. 1998). In the central nervous system (CNS) of mammals, the synthesis of both neuropeptides takes place in neuronal cells primarily found in the lateral hypothalamic area (LHA), which includes the perifornical, lateral, posterior and dorsomedial nuclei of the hypothalamus (Sakurai et al. 1998; de Lecea et al. 1998). Studies using different model systems showed that the activities of orexins are mediated by two G-protein-coupled receptors termed orexin-1 receptor (OX1R) and orexin-2 receptor (OX2R), belonging to the seven transmembrane domain receptor family. However, their activities are finely coordinated. Notably, OX1R is able to interact with OXA with high affinity. This binding is not found with OXB, for which OX1R has a low affinity. On the other hand, OX2R, although showing 64% homology with OX1R, is able to bind to both OXA and OXB with high affinity (Sakurai et al. 1998). Originally studied for their role in the central regulation of feeding (Sakurai et al. 1998) and the stimulation of arousal, cumulative findings have provided new evidence about the relevance of the orexinergic system in many physiological and pathological conditions (Jacobson et al. 2022).

On the anatomical basis, orexin neurons can be subdivided into at least two separate subpopulations, which correlate with the different brain projections and the corresponding physiological functions (Sagi et al. 2021). One subpopulation is localized in the lateral hypothalamus and sends projections to the ventral tegmental area (VTA) and nucleus accumbens (NAc), thus regulating motivation and reward (Richardson and Aston-Jones 2012). The other orexin neuronal population, residing in the perifornical and dorsomedial hypothalamus, mainly projects to the locus coeruleus (LC) and the tuberomammillary nucleus, thus modulating arousal and response to stress (Berridge et al. 2009).

In course of healthy ageing, the number of orexinergic neurons decreases in human hypothalamus by 25% from childhood to maturity (60 years old or above) (Hunt et al. 2015), consistent with reporting in rodents (Brownell and Conti 2010). As a result, the release of OXA and OXB in the CNS is reduced, leading to age-related sleep impairment, metabolic disturbances and cognitive decline. (Nixon et al. 2014).

On the other hand, the levels of orexin are increased in human cerebral spinal fluid cerebrospinal fluid (CSF) of patients with Alzheimer disease (AD) suffering from sleep disturbances (Forte et al. 2022), although there is conflicting evidence when comparing the levels in CSF with the neuroanatomical distribution (Liguori 2016). Similarly, double transgenic amyloid precursor protein/presenilin 1 (APP/PS1) mice, characterized by sleep disturbances, display significant upregulation of pre-pro-orexin mRNA and OXA in the brain compared to the age-matched wild type (Zhao et al. 2022), while orexin deficiency markedly decreased A-beta pathology in the same transgenic mice (Roh et al. 2014). Interestingly, the number of OXA neurons increases over different brain areas along the rostro-caudal axis in APP/PS1 mice compared to the wild type, suggesting that both increase and distribution of OXA neurons may play a crucial role in the progression of AD. Concordantly, the exogenous administration of OXA aggravates cognitive deficits and hippocampal synaptic plasticity impairment in the 3xTg-AD mice by increasing Aβ production and decreasing Aβ clearance through disruption of the circadian rhythm and sleep–wake cycle (Li et al. 2023). Remarkably, it has been found by our group that the interaction between OXA and endocannabinoids participates in the molecular process leading to the phosphorylation of Tau at the threonine 231 residue (pT231-Tau) (Forte et al. 2022), a sensitive and specific early marker for AD diagnosis. These findings further corroborate the involvement of OXA in the molecular mechanism of AD.

The close relationship between orexin and neurodegenerative diseases is further evident in Parkinsons’ disease (PD), in which the orexinergic system was found altered in the basal ganglia nuclei, including globus pallidus, subthalamic nucleus, *substantia nigra*, and striatum, well-established neuronal centres for the motor control (Yasui et al. 2006; Chieffi et al. 2017; Dell et al. 2013). Notably, Parkinsonian patients display significant loss of orexinergic neurons in post-mortem exams along with low orexin levels in the plasma and cerebrospinal fluid (Thannickal et al. 2007). Furthermore, research on murine models of Parkinson’s disease (PD) has shown a significant decrease in orexinergic neurons in the lateral hypothalamus (Jaggard et al., 2018). Recent studies have also highlighted OXA’s neuroprotective effects in cellular and preclinical models of PD (Liu et al. 2018b). OXA mitigated the reduction of dopaminergic neurons in the substantia nigra and restored the dopaminergic fibers in the striatum, likely through OX1R-mediated PI3K and PKC pathways, resulting in increased levels of brain-derived neurotrophic factor (BDNF) and dopamine (Liu et al. 2018b). In addition, electrophysiological analysis revealed that the administration of OXA and OXB enhanced the rate of spontaneous firing of dopaminergic neurons in the substantia nigra of rats and inhibition of OX1R and OX2R significantly decreased the firing rate of the same neurons, further corroborating the roles of the orexinergic pathway in regulating dopaminergic system (Liu et al. 2018a).

More scientific evidence highlights how the dysfunction of the orexinergic pathway is implicated in the pathogenesis of neurodegenerative diseases (*i.e.* Huntington disease, and Multiple sclerosis) other than AD and PD (Wang et al. 2021). Several therapeutic approaches based on the use of orexin receptor antagonists have been developed to rescue the sleep impairment, with some successful outcomes (Kron et al. 2023). However, the exact roles of the orexinergic system in neurodegenerative diseases and the underlying mechanisms are still to be fully understood.

## African turquoise killifish: an excellent model to study orexinergic system in ageing

In this scenario, a valid support to extend knowledge on the mechanisms of age-related regulation of the orexin system is coming from a non-mammalian model, the African turquoise killifish, *Nothobranchius furzeri*. In less than two decades this teleost fish has entered the arena of model systems validated for ageing studies. It was proposed as the shortest-lived vertebrate which can be kept under laboratory conditions and since then the scientific community has struggled to demonstrate how its compressed lifespan recapitulate the entire cycle of a vertebrate, including an accelerated ageing process (Hu and Brunet 2018). Recently, comprehensive reviews have robustly highlighted how this fish is a valuable experimental model for studying the neurobiology of ageing and age-related diseases (de Bakker and Valenzano 2023). Key relevant reported hallmarks characterizing the phenotypic spectrum of killifish brain ageing have confirmed:

- The decrease in doublecortin (DCX)-positive cells (a marker of immature neurons) in the telencephalon and posterior to the optic tectum (OT) when compared to young specimens, along with massive gliosis has been demonstrated in the radial glia of the OT (Tozzini et al. 2011);
- The loss of neuronal regenerative capacity and impossibility to fully recover from CNS injury, differently form zebrafish that maintain the neuroreparative ability albeit regenerate less efficiently at old age. This phenomenon is likely caused by the astrogliosis and the chronic brain inflammatory status, resulting in the formation of a long-term glial scar hampering the regeneration (Vanhunsel et al. 2021);
- The typical hallmarks of Parkinson disease (PD), including motor and cognitive symptoms, the formation and propagation of αSYN aggregates in Lewy bodies, and Dopamine/Norepinephrine neuronal loss (Matsui et al. 2019; Bagnoli et al. 2022);
- The decrease in proteasomal activity, leading to protein aggregation, which is driven by under-regulation of transcripts encoding components of the 19S and 20S ribosome complexes. The accumulation of proteins synthesized in excess compared to their binding partners determines a stoichiometric imbalance of protein complexes, which may be the cause of neuronal dysfunction, as also demonstrated in human patients (Sacramento et al. 2020). Strikingly, under physiological ageing Aβ Precursor Protein is neuronally synthesized and stored in the brain of the African turquoise killifish along the rostro-caudal axis with detrimental effect on neuronal survival and function (de Bakker et al. 2024). Lowering Aβ levels in mutant individuals reduces brain ageing phenotypes and mitigate age-related cognitive decline (de Bakker et al. 2024).

In light of the currently available data and considering the robustness and breadth of the degenerative phenotypes occurring in *N. furzeri*, it appears that the trajectory of brain ageing in this fish resembles that of humans with neurodegenerative diseases rather than that of healthy individuals showing normal and non-diseased brain ageing (de Bakker and Valenzano 2023). Noteworthy, the brain-associated phenotypic hallmarks of ageing seem to be diversified according to the different captive strains so far investigated (Genade and Wilcox 2021). As proof, the ZMZ1001 strain has been characterized for the physiological intraneuronal accumulation of pyroglutamated Aβ, the neurotoxic Aβ variant (de Bakker et al. 2024), thus making this strain a suitable model for AD. In contrast, a spontaneous age-dependent degeneration of dopaminergic neurons, along with and an even greater neurodegeneration of the noradrenergic neurons in the LC and dopaminergic neurons of the posterior tuberculum, was reported in the strain MZCS‐24 (Matsui et al. 2019). This strain is proposed as good model for PD despite the contrasting findings in other two strains (MZCS‐222 and MZM-0410), in which the age-related loss of dopaminergic neurons was described only in the LC and not in the dopaminergic population of the posterior tuberculum (Bagnoli et al. 2022). Altogether, these features make this model organism extremely attractive for approaching brain ageing studies and particularly to explore the mechanisms underlying the role of orexinergic system in the brain at old age. Preliminary studies have been addressed to investigate the age-related regulation of pre-pro-orexin and OXA. It was demonstrated that *i. hcrt* and OXA sequences are highly conserved in this fish species when aligned with mammalians; *ii.* the neuronal expression of *hcrt* and OXA was not restricted only to the diencephalic area, as reported in several fish species (Volkoff et al. 2016), but displayed a broader neuroanatomical distribution and neuronal projections. Most interestingly, the neuroanatomical distribution was increased in older individuals, with mRNA expressed also in the telencephalon; *iii. hcrt* hypothalamic neurons seemed to be not activated upon short-term fasting (Montesano et al. 2019) differently from neuropeptide Y; *iv.* consistently with the neuroanatomical observations, in course of ageing, *hcrt* levels showed a slight increase in the whole brain. However, published results indicate that *hcrt* was not significantly regulated in the brain of *N. furzeri* during this life stage. It is important to note that the modest increase was observed at the stage corresponding to the onset of ageing, a life stage where the typical signs of advanced ageing have not fully manifested yet. Therefore, it is likely that analyses conducted at much later stages may reveal a significant change of *hcrt* levels compared to adults, suggesting a clear impact of ageing.

These findings represent a major advancement in the study of the orexin system in vertebrates, which is typically marked by an age-associated decline (Nixon et al. 2014), occurring in parallel with physiological alterations in the regulation of obesity, sleep, and locomotor activity during ageing (Nixon et al. 2014). The age-associated increased levels of orexin in the brain of killifish likely resemble those reported in course of mammalian neurodegenerative processes (Zhao et al. 2022) and can be interpreted as a manifestation of the neurodegenerative-like phenotype identified in the brains of the old killifish brain, corroborating the hypothesis that the trajectory of brain ageing in this fish resembles that of humans with neurodegenerative diseases (de Bakker and Valenzano 2023).

Interestingly, OXA, along with several neuropeptides and metabolic hormones, has been reported to induce neurogenesis (Bakos et al. 2016). Notably, data from our group revealed that excessive OXA and downstream 2-arachidonoylglycerol/cannabinoid receptor type-1 signaling lead to the dysfunction of adult hippocampal neurogenesis, resulting in reduced plasticity and impaired pattern separation in obese mice (Forte et al. 2021). By inhibiting OXA action both plasticity and pattern separation impairment in obese mice was rescued, providing the molecular and functional mechanism to explain alterations in episodic memory in obesity. Future studies on the orexinergic system in the African turquoise killifish could add pieces of knowledge on the neurogenic activity, and particularly in the capability of new neurons to differentiate and serve specific functions.

Recently, the neuroanatomical localization of *hcrt* has been further confirmed by Bedbrook and collaborators (2023). These authors, in their proof of principle, demonstrated that CRISPR/Cas9-mediated knock-in is a powerful method in killifish to drive *hcrt* cell-type-specific expression, providing with a formidable tool for approaching new experiments oriented to disentangle the neuronal regulation of *hcrt* during vertebrate ageing (Bedbrook et al. 2023).

## African turquoise killifish *versus* zebrafish for investigating the orexinergic system

Nowadays, the most popular fish model for investigating the orexinergic system in the CNS is zebrafish, which is studied particularly for the involvement in sleep and arousal (Levitas-Djerbi and Appelbaum 2017). About 20 and 60 neurons, respectively in the larval and adult brains, compose the *hcrt* neuronal population of zebrafish, restricted to the homologous of mammalian LHA (de Lecea et al 1998; Sakurai et al. 1998). Genetic modulation of this neuronal population revealed a behavioural phenotype, *i.e.* reduction of voluntary movement during the night, clock-controlled rhythmic activity that peaks during the day, reversibility, specific posture, increased arousal thresholds, and sleep rebound post-sleep deprivation, all criteria used to characterize zebrafish sleep (Zada and Appelbaum 2020; Elbaz et al. 2013).

From a functional perspective, our research group has demonstrated that the evolutionary crosstalk between the OXA and endocannabinoid systems is conserved in the zebrafish brain. This is demonstrated by the fact that OX-A induces 2-AG biosynthesis, likely mediated by OX-2R (Imperatore et al. 2018). Additionally, the overlapping neuroanatomical distribution of orexin and endocannabinoid receptors supports the hypothesis that orexin/endocannabinoid signaling interactions in the adult zebrafish brain are similar to those observed in corresponding regions of the mammalian brain, where the two receptors are contiguous (Imperatore et al. 2018) The single-cell analysis strategy in zebrafish larvae identified hundreds of novel *Hcrt*-neuron-specific transcripts mainly involved in regulation of metabolism, sleep, synaptogenesis, and synaptic plasticity (Yelin-Bekerman et al. 2015). Similar approach was taken also in mammals and revealed a genetic heterogeneity of the orexin subpopulations (Iyer et al. 2018). However, a precise overview of such heterogeneity is limited due to the anatomical organization of the orexinergic subpopulations, which are intermingled and not completely segregated (Iyer et al. 2018). Interestingly, recent single-cell profiling of glutamatergic and GABAergic cell types in the zebrafish hypothalamic periventricular zone revealed evolutionary conserved and divergent hypothalamic cell types between fish and mouse. Subsets of glutamatergic *Hcrt* neurons in both larval and adult zebrafish expressed also *neuropeptide VF (Npvf)*, a specific gene encoding a sleep-regulating neuropeptide. The genetic profile, activity, and neurite processing of the neuronal subpopulation that co-expresses both *Hcrt* and *Npvf* differ from other *Hcrt* neurons (Sagi et al. 2024), suggesting that the heterogeneity of *Hcrt* neurons enables multifunctionality, such as consolidation of both wake and sleep by the *Hcrt*- and *Npvf*-releasing neuronal subpopulation. Interestingly, orexinergic system is hypothesized to play a crucial role in regulating the circadian rhythms of sleep and wakefulness. Although, it is still unclear whether these rhythms are controlled by the orexin peptides themselves or by other signaling molecules released by these neurons, such as glutamate or dynorphin. Recent gene expression profiling by transcriptomic data supports this hypothesis confirming that the expression profile of 22 circadian genes was altered in the ageing CNS of African turquoise killifish, when the expression of orexinergic system is changed. Among the key genes, Per2, a critical circadian rhythm (CR) regulator involved in neuronal plasticity and wakefulness maintenance, was found to be upregulated in aged African turquoise killifish and other examined species. The latter result is particularly relevant as, in mammals, Per2 interacts with the orexinergic system to regulate the sleep–wake cycle (Ma et al. 2016), suggesting an evolutionarily conserved molecular mechanism. Overall, these findings strongly suggest that circadian dysregulation might be linked to sleep disorders and neurodegenerative dysfunctions (Ma et al. 2016) and further corroborate the use of this fish species as a valuable model for investigating the relationship between CR decline and orexinergic system, as already established in zebrafish and mammals. Most remarkably, the circadian activity, as reflected by locomotor activity rhythms, was investigated in a very close-related species, *Nothobranchius kortusae* (Lucas-Sánchez et al. 2011). Twenty-four-week-old fish exhibited a robust, typically diurnal rhythm, which deteriorated with age, resulting in a loss of regularity and amplitude, and a marked fragmentation of locomotor activity.These observations were sustained by the accurate phenotypic characterization of the typical hallmarks of ageing, such as weight and colouration losses, caudal fin degradation, cataractogenesis and a high peroxidation index (Lucas-Sánchez et al. 2011). Geriatric animals of *Nothobranchius kortusae* improved the regularity, fragmentation and amplitude of the rest-activity rhythm and sleep efficiency upon melatonin treatment, reinforcing the hypothesis that *Nothobranchius* species are suitable model for studying the ageing of the circadian system and the restorative effect of chronobiotic substances (Lucas-Sánchez et al. 2013). The latter approach can also be pursued in the African turquoise killifish, to compare the physiological response in animals belonging to different strains (Genade and Wilcox 2021).

The availability of a knock-in model having *hcrt* fluorescent neurons, such as the case of the African turquoise killifish, offers the unique possibility to conduct studies at single cell level as well as characterize the neuroanatomy of orexinergic neuronal population(s) in course of ageing. As a proof, this knock-in model could enhance the research on the interconnection between orexinergic neurons and neuronal population of the LC in the ageing of the African turquoise killifish. The loss of orexinergic neurons in the hypothalamus and LC was associated with a significant decrease in tyrosine hydroxylase (TH) mRNA in the LC, despite no change was reported in number of TH-immunoreactive neurons, in the ageing rhesus macaque (Downs et al. 2006), thus suggesting that age-related decrease in excitatory orexin innervation to the noradrenergic LC may contribute to the aetiology of sleep/arousal impairment in the elderly.

**Fig. 1:**
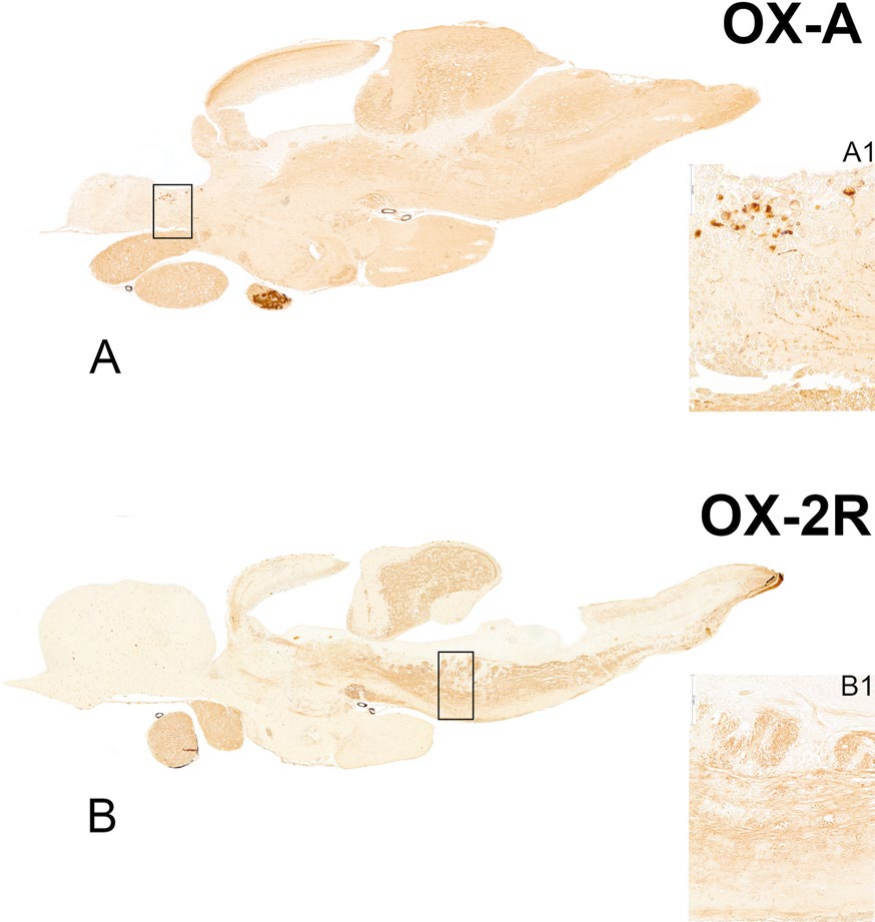
Overview of the distribution pattern of OXA and OX2R in whole encephalon of adult African turquoise killifish. **A**. Mosaic of parasagittal section of the brain depicting immunoreactivity to OXA in neurons of the preoptic and hypothalamic area and in the hypophysis. Abundance neuronal projections toward the optic tect and the rhomboencephalic region. A1. Higher magnification of OXA positive neuronal bodies and projections in the preoptic area. **B**. Mosaic of parasagittal section of the brain depicting immunoreactivity to OX2R in neuronal projections toward the optic tect and the rhomboencephalic region. B1. Higher magnification of OX2R immunoreactive fibers in the raphe nuclei. Scale bar A-B: 1.2 mm

Recently, two different studies have been addressed to characterize the age-dependent dopaminergic population of LC in different strains of the African turquoise killifish: a 30% of reduction of dopaminergic neurons characterized by protein aggregates within the cytoplasm in MZCS-222 and MZM-0410 (Bagnoli et al. 2022) and up to 80% of neuronal loss in old brain of individuals belonging to MZCS-24 strain (Matsui et al. 2019). Evidence in rodents highlighted how the LC is activated following whole brain exposure to OXA, which may thereby represent a key target brain nucleus to consider in the validation of disease mechanisms that could be modulated by orexinergic pharmacology (Holton et al. 2020). Our neuroanatomical observations have confirmed that the orexin neuronal fibres massively project to the brainstem, including the LC, of the African turquoise killifish and that OX2R is abundantly distributed in the brainstem (Fig. 1), suggestive of an evolutionary conserved pattern. More recently, a comparative neuroanatomical study has demonstrated that in the mouse brain the highest expression of *Hcrtr1* mRNA was in the LC whereas *Hcrtr2* mRNA was mostly in the lateral hypothalamus and the noradrenergic neurons of the human LC showed high signals for both *HCRTR1* (71.7%) and *HCRTR2* (81.5%) mRNA. The observed distribution pattern in mouse and human brains is consistent with the involvement of the orexin system in arousal and the sleep/wake cycle in both species, despite certain variations in receptor subtype expression profiles (Mir et al. 2024). Altogether these data corroborate and pave the way for new experimental designs addressed to disentangle the neuronal mechanisms regulating the orexinergic system in aged brains of vertebrates.

## Conclusions

Considering current research, the African turquoise killifish stands out as a valuable model for studying ageing and the modulation of the orexinergic system, due to its short lifespan and the opportunity it offers to track neurobiological changes over time. A particularly intriguing feature of this species is the widespread and dispersed distribution of orexinergic neurons, which differ not only from mammals, but also from other vertebrate models such as zebrafish, where the orexinergic neuron population is more confined to specific brain regions (de Lecea et al 1998; Sakurai et al. 1998). This distinction may represent an evolutionary adaptation to different life cycles and environments, suggesting that orexin might serve variable functions across species based on their evolutionary histories.

In zebrafish, the orexinergic system plays a key role in sleep regulation, exhibiting behaviors that parallel those seen in rodents, with clear effects on sleep–wake cycle modulation (Zada and Appelbaum 2020; Elbaz et al. 2013;). However, unlike mammals, zebrafish do not display cataplexy associated with orexin deficiency, indicating that the evolutionary trajectory of the orexinergic system may have followed divergent pathways, despite the system is evolutionarily well-conserved already in Metazoa (Alzugaray et al. 2019) and elasmobranch (Hara et al. 2018). However, it should be considered that orexins and their receptors are implicated in vital functions including fertility, appetite, digestion other than sleep in mammals, as well as osmolarity and gastrointestinal control in teleost fishes (Hara et al. 2018). Indeed, a recent study reported lack of functional hypocretin system in the fish species *Chromobotia macracanthus* (family *Botiidae*) (Bitsikas et al. 2024), characterized by absence of cataplexy episodes or sudden behavioral arrest. In contrast, a sharp difference in regulation of the orexinergic system has been investigated in the brain of fish belonging to the surface and cave populations of *Astyanax Mexicanus*. The overexpressed levels of hypocretin/orexin in the brain of this teleost species*,* three-fold higher compared to the surface fish, paralleled by the increased number of Hypocretin/Orexin (HCRT)-positive hypothalamic neurons, may explain the sleep loss observed in this species. Indeed, pharmacological or genetic inhibition of HCRT signaling, alongside manipulations such as the ablation of the lateral line or starvation, known to selectively promote sleep in cavefish, determined sleep increase in this species. This further supports the conserved role of HCRT in sleep regulation.

In conclusion, future research on the African turquoise killifish, with its accelerated ageing and unique orexinergic neuron distribution, could yield valuable insights into the evolutionary roles of orexin and its regulation of sleep across the ageing process, further enhancing our understanding of evolutive aspects of orexinergic system.

## Data Availability

No datasets were generated or analysed during the current study.
